# Neuroblastoma in Childhood: Biological Insights, Risk Stratification, and Advances in Multimodal Therapy

**DOI:** 10.3390/jcm15031101

**Published:** 2026-01-30

**Authors:** Amina De Bona, Martina Barbieri, Nicole Rinaldi, Susanna Esposito

**Affiliations:** Pediatric Clinic, Department of Medicine and Surgery, University of Parma, 43126 Parma, Italy; amina.debona@unipr.it (A.D.B.); martina.barbieri@unipr.it (M.B.); nicole.rinaldi@unipr.it (N.R.)

**Keywords:** neuroblastoma, risk stratification, MYCN amplification, immunotherapy, targeted therapy, molecular profiling, precision medicine

## Abstract

Neuroblastoma is the most common extracranial solid tumor of childhood and remains a leading cause of cancer-related mortality in pediatric patients. Characterized by marked clinical and biological heterogeneity, the disease ranges from spontaneously regressing tumors in infants to highly aggressive, treatment-resistant malignancies in older children. Advances in molecular biology and genomics have significantly improved understanding of neuroblastoma pathogenesis, revealing the critical role of genetic and epigenetic alterations—such as *MYCN* amplification, *ALK* mutations, and chromosomal aberrations—in disease behavior and prognosis. Contemporary risk stratification systems now integrate clinical, biological, and molecular features to guide therapy more precisely. Management strategies have evolved toward risk-adapted, multimodal approaches. Low- and intermediate-risk patients often achieve excellent outcomes with surgery alone or limited chemotherapy, whereas high-risk neuroblastoma requires intensive multimodal treatment including induction chemotherapy, surgical resection, high-dose chemotherapy with autologous stem cell rescue, radiotherapy, and maintenance therapy. The incorporation of immunotherapeutic approaches, particularly anti-GD2 monoclonal antibodies, has significantly improved survival in high-risk disease. Emerging therapies such as targeted agents, radiopharmaceuticals, and cellular immunotherapies are further expanding the therapeutic landscape. Despite these advances, high-risk and relapsed neuroblastoma remain associated with substantial morbidity and mortality. Ongoing challenges include treatment resistance, long-term toxicity, and disparities in access to advanced therapies. Continued progress will depend on integrating molecular profiling into clinical decision-making, refining risk-adapted treatment strategies, and expanding international collaborative research efforts. This narrative review summarizes current knowledge on neuroblastoma epidemiology, biology, staging, and treatment, highlighting recent advances and future directions aimed at improving outcomes for affected children.

## 1. Background

Neuroblastoma is the most common extracranial solid malignancy of childhood, accounting for approximately 7–10% of all pediatric cancers [1,2]. It most frequently arises from the adrenal medulla or along the paravertebral sympathetic chain. The disease predominantly affects young children, with the majority of diagnoses occurring before the age of five and a median age at diagnosis of approximately 17 months [3]. Despite representing a relatively small proportion of childhood cancers, neuroblastoma is responsible for nearly 15% of all pediatric oncology-related deaths.

Clinical outcomes vary widely according to biological and clinical risk stratification. While children with low- or intermediate-risk disease achieve excellent long-term outcomes—with 5-year overall survival (OS) rates approaching 95%—the prognosis for high-risk neuroblastoma remains poor, with survival rates below 50% despite intensive multimodal therapy [4,5]. This striking disparity underscores the urgent need for improved risk stratification and more effective, biology-driven therapeutic approaches for aggressive disease.

Neuroblastoma originates from neural crest-derived sympathoadrenal progenitor cells. During embryogenesis, neural crest cells migrate from the dorsal neural tube, undergoing epithelial-to-mesenchymal transition before differentiating into components of the sympathetic nervous system and adrenal medulla. A subset of Schwann cell precursors—often referred to as “bridge cells”—can differentiate either into chromaffin cells or into sympathoadrenal neuroblasts, which continue their maturation postnatally, typically up to the age of three years [2]. Disruption of these tightly regulated developmental processes is believed to underlie tumorigenesis.

Although the precise timing and cellular origin of malignant transformation remain incompletely defined, accumulating evidence indicates that neuroblastoma heterogeneity reflects both the developmental stage at which oncogenic events occur and the degree of cellular plasticity within the tumor microenvironment [6]. Transcriptomic analyses have shown that low-risk tumors share molecular features with more differentiated neuroblasts or postnatal chromaffin cells, whereas high-risk tumors resemble immature adrenal progenitors or bridge-cell populations. These findings suggest that aggressive neuroblastomas may arise either from early oncogenic insults during embryogenesis or from a failure of postnatal differentiation, with up to one-third of cases potentially originating during the first trimester of gestation [2].

Genetic and chromosomal alterations play a central role in determining tumor behavior, prognosis, and treatment response. Despite this, only 1–2% of neuroblastoma cases are hereditary, with a documented positive family history [2]. Consequently, germline predisposition testing is currently recommended only for selected patients, particularly those with familial clustering or pathogenic variants identified in multiple unrelated individuals.

Among known predisposition genes, activating germline mutations in the *ALK* proto-oncogene account for approximately 50–75% of hereditary neuroblastoma cases [5,7]. *ALK* signaling plays a key role in sympathoadrenal development, and its aberrant activation synergizes with *MYCN* oncogenic signaling, contributing to tumor aggressiveness and poor prognosis [8]. *MYCN* amplification, present in approximately 20% of all neuroblastomas and up to 40% of high-risk cases, remains one of the strongest adverse prognostic factors, conferring resistance to conventional chemotherapy and radiotherapy [9].

Advances in molecular characterization have led to significant therapeutic innovations. Identification of *ALK* alterations has enabled the development of targeted tyrosine kinase inhibitors; *MYCN*-driven oncogenesis has prompted exploration of indirect targeting and differentiation-based therapies; and immunotherapeutic approaches such as anti-GD2 monoclonal antibodies and ^131I-MIBG therapy have become integral components of high-risk disease management. Emerging strategies—including chimeric antigen receptor (CAR) T-cell therapy, pathway-specific inhibitors, and combination regimens targeting tumor plasticity—are currently under active investigation [10,11,12].

In addition to genetic predisposition, several congenital syndromes (including rapid-onset obesity with hypothalamic dysfunction, hypoventilation, and autonomic dysregulation [ROHHAD] syndrome, Weaver, Costello, and Beckwith–Wiedemann syndromes) are associated with an increased risk of neuroblastoma, warranting tailored surveillance strategies [7]. Germline genetic testing is recommended for patients with a positive family history, multifocal or bilateral disease (approximately 4% of cases), or when tumor sequencing reveals pathogenic or likely pathogenic variants that may represent germline predisposition [13,14]. Identification of such variants has important implications for family counseling and surveillance.

From a preventive and surveillance perspective, imaging plays a central role. Approximately 80% of neuroblastomas arise along the paraspinal sympathetic chain or adrenal medulla and can be detected by ultrasonography, while thoracic and cervical tumors (approximately 20%) require cross-sectional imaging. Moreover, because nearly 90% of neuroblastomas secrete catecholamines, measurement of urinary vanillylmandelic acid (VMA) and homovanillic acid (HVA) remains a valuable, non-invasive diagnostic and monitoring tool [7].

This narrative review aims to synthesize current evidence on the epidemiology, biological foundations, and risk stratification of neuroblastoma, as well as to highlight advances in therapeutic strategies and emerging research directions. By integrating molecular insights with clinical practice, we aim to provide a comprehensive framework to support precision medicine approaches and improve outcomes for children affected by this complex disease. While several high-quality reviews have recently summarized individual aspects of neuroblastoma biology, risk stratification, or treatment advances, the present narrative review aims to provide a unifying and integrative perspective that bridges molecular insights, contemporary risk classification systems, therapeutic innovation, and long-term survivorship considerations within a single clinical framework. Unlike prior reviews that focus predominantly on either biological mechanisms or treatment modalities in isolation, this work emphasizes the continuum of care, from tumor origin and molecular drivers to therapy-related toxicity, survivorship, and quality of life. A particular focus is placed on emerging challenges that remain insufficiently addressed in the literature, including mechanisms of resistance to targeted therapies, the neurodevelopmental and psychosocial consequences of intensive treatment during critical windows of brain maturation, and the long-term burden of late effects in an expanding survivor population. By highlighting these unresolved controversies and knowledge gaps, the review seeks to inform future translational research, refine risk-adapted therapeutic strategies, and support the development of comprehensive, multidisciplinary survivorship care models tailored to the evolving needs of children with neuroblastoma.

## 2. Methods

This narrative review was conducted to provide a comprehensive and up-to-date overview of neuroblastoma, with particular emphasis on epidemiology, underlying biological mechanisms, risk stratification, and current as well as emerging therapeutic strategies. A narrative approach was deliberately chosen to allow for the integration of heterogeneous sources of evidence—including clinical trials, translational research, and international consensus guidelines—and to contextualize evolving concepts within the rapidly advancing field of pediatric oncology.

A structured literature search was performed using major biomedical databases, including PubMed/MEDLINE, Scopus, and Web of Science, covering publications available up to March 2025. The search strategy combined Medical Subject Headings (MeSH) and free-text terms related to neuroblastoma, including but not limited to “neuroblastoma”, “pediatric cancer”, “risk stratification”, “*MYCN*”, “*ALK*”, “immunotherapy”, “MIBG”, “CAR-T”, “genetic predisposition”, and “treatment outcomes”. Additional relevant publications were identified through manual screening of reference lists from key articles and recent review papers.

Inclusion criteria comprised peer-reviewed articles addressing childhood neuroblastoma that provided data or expert insight into epidemiology, tumor biology, molecular and genetic features, staging and risk classification systems, therapeutic strategies, or clinical outcomes. Priority was given to randomized controlled trials, prospective and retrospective cohort studies, large cooperative group trials, systematic reviews, meta-analyses, and international clinical practice guidelines. Translational and preclinical studies were included when they offered clinically relevant insights into disease mechanisms or therapeutic innovation. Case reports and small case series were selectively included when describing rare clinical presentations, uncommon complications, or emerging treatment approaches not yet supported by larger studies.

Exclusion criteria included non-peer-reviewed articles, conference abstracts without full-text availability, editorials lacking original data or substantive expert synthesis, studies unrelated to pediatric neuroblastoma, and publications with insufficient methodological detail. Articles focusing exclusively on adult neuroblastoma without relevance to pediatric disease were excluded. Only manuscripts published in English were considered.

Data extraction focused on epidemiology, clinical presentation, staging and risk classification systems, molecular and genetic characteristics, therapeutic strategies, and outcome measures. Particular emphasis was placed on studies with high methodological quality, well-defined patient populations, and relevance to contemporary clinical practice. Where available, consensus statements and internationally accepted classification systems were used to enhance consistency and clinical applicability.

Given the narrative design of this review, no formal systematic review protocol or meta-analytic methods were applied. Instead, the selected literature was synthesized qualitatively to provide an integrated and critical overview of current knowledge, to highlight areas of ongoing debate, and to identify gaps warranting further investigation.

## 3. Epidemiology

Neuroblastoma is predominantly diagnosed in early childhood, with the majority of cases occurring before 5 years of age and a median age at diagnosis of approximately 17 months [3]. Marked disparities in epidemiology and clinical outcomes have been documented across different ethnic and geographic populations. Children of African and Native American ancestry exhibit a higher prevalence of high-risk disease and significantly poorer survival outcomes compared with children of European descent. In particular, children of African ancestry demonstrate higher rates of relapse beyond two years from diagnosis, even after adjustment for clinical and biological risk factors, suggesting an increased resistance to standard therapeutic approaches.

Distinct outcome patterns have also been reported among Asian populations, in whom overall survival appears inferior compared with Caucasian cohorts, despite comparable event-free survival rates. In contrast, Hispanic children show outcomes largely comparable to those observed in Caucasian populations [15].

Recent analyses derived from the Global Burden of Disease study have provided a comprehensive overview of worldwide trends in childhood neuroblastoma from 1990 to 2021, examining incidence, mortality, and disease burden as measured by years of life lost and years lived with disability [16]. These data reveal a modest global increase in incidence over time, accompanied by rising absolute numbers of deaths and disability, particularly in low- and middle-income countries. Regions such as South Asia and sub-Saharan Africa continue to experience disproportionately high mortality rates, reflecting persistent disparities in access to early diagnosis, specialized care, and advanced treatment modalities. In contrast, high-income countries have experienced stabilization or even a decline in mortality rates.

Age-related disparities are particularly evident, with the greatest differences in incidence and mortality observed among younger children. These findings underscore the critical influence of socioeconomic conditions, healthcare infrastructure, and timely access to diagnosis and treatment on outcomes in early childhood.

An additional emerging trend is the shifting nature of disease burden. In many regions—especially those with lower or middle sociodemographic indices—the increase in years lived with disability has outpaced reductions in years of life lost. This reflects the success of modern therapeutic strategies in prolonging survival but also highlights the growing population of survivors living with long-term sequelae, including neurological, endocrine, and cardiovascular complications. This epidemiological transition underscores the need for comprehensive survivorship care models that extend beyond disease eradication to address long-term morbidity and quality of life [17].

As survival rates continue to improve, an increasing proportion of children with neuroblastoma enter long-term survivorship, often carrying a substantial burden of late effects, including neurological, cognitive, endocrine, and psychosocial complications. This issue becomes particularly salient as survivors transition into adolescence, a critical developmental period characterized by profound brain maturation, heightened neural plasticity, and increased sensitivity to social and environmental influences. During this window, dynamic interactions between neurobiological development and psychosocial context play a pivotal role in shaping cognitive function, emotional regulation, and mental health. In this regard, recent work by Liu et al. [18] provides a contemporary neurobiological framework by delineating neural, cognitive, and psychopathological signatures associated with environmental exposures during early adolescence. Integrating such developmental neuroscience perspectives is essential for understanding how cancer-related neurotoxicity, chronic stress, and altered social experiences may interact with ongoing brain development in pediatric cancer survivors. Collectively, these considerations reinforce the need for longitudinal, multidisciplinary survivorship care models, as highlighted in the Epidemiology section, that extend beyond disease control to include structured, lifelong monitoring of cognitive trajectories, mental health, and quality of life from adolescence into adulthood.

## 4. Clinical Presentation

Approximately 65% of neuroblastomas arise from the adrenal medulla or the paravertebral sympathetic and celiac ganglia, frequently presenting as an abdominal mass and/or symptoms related to compression of adjacent organs [19,20]. In about 5% of cases, the tumor originates from the stellate ganglion, manifesting as a laterocervical mass that may be associated with Horner syndrome—characterized by ptosis, miosis, and anhidrosis—which can occasionally represent the first clinical sign of disease [21].

Tumors arising from mediastinal sympathetic ganglia account for approximately 20% of cases and may present with respiratory symptoms due to airway compression. In infants, abdominal distension caused by large subdiaphragmatic tumors may be a prominent presenting feature.

At diagnosis, metastatic dissemination is observed in approximately 40% of patients, most commonly involving the bone marrow and skeletal system. These patients often present with bone pain, anemia, thrombocytopenia, fever, and general systemic symptoms. Metastases to lymph nodes, liver, and skin are less frequent, whereas pulmonary and central nervous system involvement are rare and occur predominantly in adolescents [22].

Ocular manifestations, particularly proptosis and periorbital ecchymosis (“raccoon eyes”), are characteristic signs of advanced disease and reflect retro-orbital metastatic involvement [23]. Cutaneous metastases, typically presenting as bluish subcutaneous nodules, are more commonly observed in infants and represent a distinctive clinical feature of disseminated disease in this age group [24].

Tumors originating from paraspinal sympathetic ganglia may extend through the neural foramina into the spinal canal, resulting in spinal cord compression. This condition constitutes an oncologic emergency and requires prompt intervention—often combining corticosteroids, chemotherapy, and/or surgical decompression—to prevent irreversible neurological damage [25].

Rarely, neuroblastoma may present with severe secretory diarrhea due to ectopic production of vasoactive intestinal peptide (VIP). Additional uncommon manifestations include hypertension and headache related to excessive catecholamine secretion [20,26].

Paraneoplastic neurological syndromes, such as opsoclonus–myoclonus–ataxia syndrome (OMAS), occur in approximately 2–3% of patients. These immune-mediated manifestations are characterized by chaotic eye movements, myoclonus, ataxia, and behavioral disturbances. Although children with OMAS often have a favorable oncologic prognosis, neurological sequelae may persist or emerge after tumor treatment, frequently requiring long-term immunomodulatory therapy and neurodevelopmental follow-up [27,28].

## 5. Staging Systems and Risk Grouping

Accurate staging of neuroblastoma relies on comprehensive imaging evaluation. Ultrasonography (US) is typically the first-line diagnostic modality, particularly in infants and young children, and is especially useful for identifying tumors arising in the neck, abdomen, or pelvis. Common ultrasonographic features include heterogeneous masses with internal calcifications and encasement or displacement of adjacent vascular structures [29].

Although computed tomography (CT) provides excellent anatomical detail, the current gold standard for diagnostic imaging is the combined use of magnetic resonance imaging (MRI)—particularly diffusion-weighted imaging (DWI)—and functional nuclear medicine studies with meta-[^123I]iodobenzylguanidine (MIBG) scintigraphy. MRI allows superior soft-tissue contrast, precise assessment of intraspinal extension, and evaluation of vascular and organ involvement, while avoiding ionizing radiation. MIBG scintigraphy, which exploits the norepinephrine transporter expressed by most neuroblastoma cells, is positive in approximately 90% of cases and enables sensitive detection of both primary tumors and metastatic disease. When combined with single-photon emission computed tomography (SPECT), MIBG imaging provides integrated functional and anatomical information that enhances lesion localization and staging accuracy [30].

Overall, high-quality imaging is essential not only for accurate disease staging but also for surgical planning, risk stratification, and treatment response assessment, playing a central role in the multidisciplinary management of neuroblastoma.

### 5.1. International Neuroblastoma Staging System (INSS)

The International Neuroblastoma Staging System (INSS) was introduced in 1986 and subsequently adopted by the Children’s Oncology Group (COG) as a standardized framework for disease classification [13]. The INSS is a postsurgical staging system based on the extent of tumor resection, the relationship of the tumor to midline structures, regional lymph node involvement, and the presence or absence of distant metastases. According to these criteria, neuroblastoma is classified into stages 1, 2A, 2B, 3, 4, and 4S.

A distinctive feature of the INSS is the inclusion of stage 4S, a category unique to neuroblastoma. This stage applies to infants younger than 12 months who present with a localized primary tumor (stage 1 or 2) accompanied by limited metastatic spread confined to the skin, liver, and/or bone marrow. Despite the presence of metastatic disease, stage 4S neuroblastoma is associated with a remarkably favorable prognosis and, in many cases, spontaneous regression.

The biological mechanisms underlying spontaneous regression remain incompletely understood. It is hypothesized that the developmental maturation of neuroblasts, immune-mediated tumor clearance, or apoptosis triggered by unfavorable microenvironmental conditions may contribute to tumor involution. Population-based screening programs using urinary catecholamine measurements in infants have provided indirect evidence for this phenomenon, revealing that a substantial proportion of neuroblastomas—particularly in children younger than 12 months—undergo spontaneous regression and therefore remain clinically undetected. These observations underscore the unique natural history of neuroblastoma and highlight fundamental biological differences between this tumor and most other pediatric malignancies [31].

### 5.2. International Neuroblastoma Risk Group Staging System (INRGSS)

In 2009, the International Neuroblastoma Risk Group (INRG) introduced a revised staging system designed to overcome the limitations of the surgically based INSS and to enable pre-treatment risk stratification [32,33,34]. Unlike the INSS, which relies on postoperative findings, the INRG Staging System (INRGSS) classifies disease before any surgical intervention, allowing for more accurate treatment planning and prognostic assessment based on initial tumor characteristics.

The INRGSS categorizes tumors as either localized (L1 or L2) or metastatic (M or MS), with classification primarily determined by the presence or absence of image-defined risk factors (IDRFs) identified on diagnostic imaging. IDRFs describe specific anatomic features—such as encasement of major vessels or invasion of vital structures—that are associated with increased surgical complexity, higher risk of incomplete resection, and poorer clinical outcomes [32].

Stage L1 tumors are localized to a single body compartment (neck, chest, abdomen, or pelvis) and show no IDRFs, indicating that complete surgical resection is feasible with low operative risk. In contrast, stage L2 tumors are also locoregional but exhibit one or more IDRFs, reflecting a higher likelihood of surgical morbidity and a more aggressive clinical course.

Metastatic disease is classified as stage M when distant metastases are present, regardless of the primary tumor site. A distinct category, stage MS, applies to infants younger than 18 months who exhibit metastatic spread limited to the skin, liver, and/or bone marrow, with bone marrow involvement restricted to less than 10% of nucleated cells [33,34]. Despite the presence of metastases, stage MS disease is associated with a relatively favorable prognosis and, in some cases, spontaneous regression.

Overall, the INRG staging system represents a major advance in neuroblastoma classification by enabling risk-adapted therapeutic strategies, improving prognostic accuracy, and facilitating uniform patient stratification across international clinical trials.

The assessment of IDRFs extends beyond simple classification into L1 or L2 disease and plays a central role in evaluating surgical feasibility and operative risk. Careful analysis of IDRFs is essential for preoperative planning, as their presence correlates strongly with an increased likelihood of intraoperative complications, incomplete tumor resection, and postoperative morbidity. In general, a greater number of IDRFs is associated with progressively lower rates of complete surgical excision and less favorable outcomes.

The identification of two or more IDRFs at diagnosis is considered a critical threshold and should prompt consideration of neoadjuvant chemotherapy prior to surgical intervention. Preoperative tumor shrinkage achieved through chemotherapy can reduce surgical risk, facilitate safer resection, and improve overall outcomes. Consequently, IDRF assessment has become a cornerstone of contemporary neuroblastoma management, guiding both the timing and strategy of surgical intervention within a multidisciplinary treatment approach [35,36].

### 5.3. International Neuroblastoma Risk Group—Pre-Treatment Risk Group Classification

The International Neuroblastoma Risk Group (INRG) pre-treatment risk classification system [37] integrates disease stage (L1, L2, M, or MS) with key biological and clinical prognostic factors, including age at diagnosis, tumor histology, degree of differentiation, *MYCN* amplification status, 11q chromosomal aberrations, and tumor ploidy.

By combining these variables, the INRG system stratifies patients into 16 distinct risk categories, which are subsequently consolidated into four overarching risk groups: very low, low, intermediate, and high risk. This comprehensive stratification enables a more accurate prediction of prognosis and provides a standardized framework for assigning risk-adapted therapeutic strategies across international clinical trials. The INRG classification has therefore become a cornerstone in modern neuroblastoma management, facilitating both individualized treatment planning and meaningful comparison of outcomes across studies [32,37].

## 6. Therapeutic Strategies

According to the International Neuroblastoma Risk Group (INRG) classification, patients are stratified into three principal risk categories—low, intermediate, and high risk—based on a combination of clinical, pathological, and biological features.

Children with low- and intermediate-risk neuroblastoma generally experience excellent outcomes. In these patients, treatment strategies involving surgery alone or in combination with low-intensity chemotherapy achieve long-term event-free survival rates exceeding 90% [38].

In contrast, high-risk neuroblastoma continues to represent a major therapeutic challenge. Despite substantial advances in multimodal treatment approaches—including intensive chemotherapy, surgical resection, radiotherapy, high-dose chemotherapy with autologous stem cell transplantation, and immunotherapy—long-term survival remains limited, with current rates ranging between 40% and 60% [39]. These outcomes underscore both the progress achieved over recent decades and the ongoing need for innovative and more effective therapeutic strategies for this high-risk population.

### 6.1. Treatment of Low- and Intermediate-Risk Neuroblastoma

Non-high-risk neuroblastoma represents more than half of newly diagnosed cases and encompasses a biologically and clinically heterogeneous group of patients. These tumors are typically characterized by localized or limited metastatic disease and the absence of *MYCN* amplification. Overall outcomes are highly favorable, and treatment strategies are tailored to minimize therapy-related toxicity while maintaining excellent survival rates.

For patients with localized, resectable tumors and favorable biological features, surgical resection alone is often curative. In such cases, chemotherapy is reserved for relapse or disease progression following surgery [40]. Very low-risk tumors, such as prenatally detected adrenal neuroblastomas or small masses identified within the first months of life, demonstrate an exceptionally benign clinical course. These lesions frequently undergo spontaneous regression, and active surveillance with serial ultrasonography every 6–8 weeks is generally recommended. Surgical intervention is reserved for tumors that demonstrate progressive growth or fail to regress. Following resection, imaging surveillance—typically every three months during the first year and every six months during the second year—is advised to monitor for recurrence [41,42].

Children with stage MS (formerly 4S) disease represent a distinct subgroup. Although these patients may present with extensive disease manifestations such as massive hepatomegaly, respiratory compromise, liver dysfunction, or coagulopathy, many experience spontaneous tumor regression. However, when life-threatening symptoms are present, prompt therapeutic intervention is required to prevent morbidity or mortality [43].

Intermediate-risk neuroblastoma is managed with a combination of moderate-intensity chemotherapy and surgical resection. According to the COG A3961 protocol, patients are stratified into two subgroups based on biological features: those with favorable biology (favorable histology and DNA index > 1) and those with unfavorable biology (unfavorable histology and/or DNA index ≤ 1). Patients in the favorable group typically receive four cycles of chemotherapy, whereas those in the unfavorable group receive up to eight cycles. Despite differences in treatment intensity, overall survival rates in both subgroups exceed 95%, underscoring the effectiveness of risk-adapted therapeutic strategies in this population [36].

### 6.2. Treatment of High-Risk Neuroblastoma

**Figure 1:**
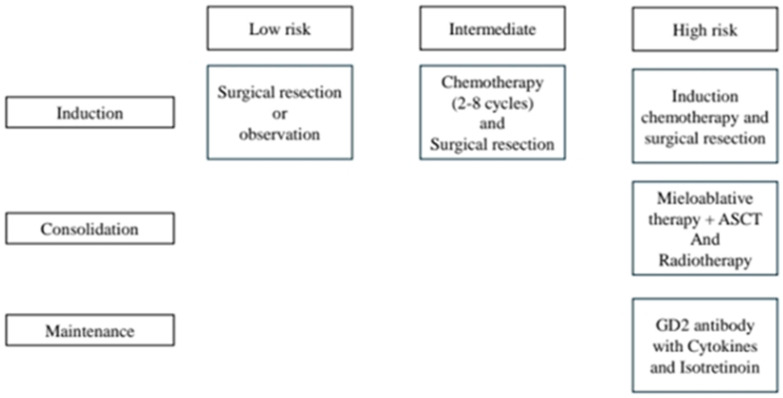
Treatment stages in neuroblastoma based on risk groups. Adapted from ref. [44].

Children with high-risk neuroblastoma continue to represent one of the most therapeutically challenging groups in pediatric oncology (Figure 1). Despite major advances in multimodal treatment strategies, outcomes for these patients remain suboptimal, with a substantial risk of relapse and treatment-related morbidity.

Management of high-risk neuroblastoma follows a multimodal, sequential approach consisting of three main therapeutic phases. The induction phase involves intensive multi-agent chemotherapy, often combined with surgical resection of the primary tumor, with the aim of achieving maximal tumor cytoreduction. This is followed by a consolidation phase, which includes high-dose myeloablative chemotherapy supported by autologous stem cell transplantation (ASCT) and locoregional radiotherapy to eradicate residual disease. Finally, the maintenance phase is designed to eliminate minimal residual disease and reduce relapse risk, typically employing differentiation therapy with isotretinoin in combination with immunotherapeutic agents targeting tumor-associated antigens, such as anti-GD2 monoclonal antibodies [45].

Despite this aggressive, multimodal approach, long-term survival rates for high-risk neuroblastoma remain limited, underscoring the need for continued refinement of existing strategies and the development of novel, biology-driven methods.

#### 6.2.1. Induction Phase

The induction phase represents the initial and critical component of treatment for patients with high-risk neuroblastoma, aiming to achieve maximal tumor cytoreduction and control of metastatic disease prior to consolidation therapy. Current induction regimens are based on intensive multi-agent chemotherapy combinations that have demonstrated efficacy in achieving early disease control.

The COG protocol typically consists of six cycles of induction chemotherapy. This includes two initial cycles of topotecan combined with cyclophosphamide, followed by alternating cycles of cisplatin plus etoposide and cyclophosphamide, doxorubicin, and etoposide in cycles three through six [46]. This regimen is designed to maximize tumor cytoreduction while maintaining an acceptable toxicity profile.

In Europe, the International Society of Paediatric Oncology European Neuroblastoma Group (SIOPEN) has adopted the rapid COJEC regimen, which consists of eight cycles of cisplatin, carboplatin, cyclophosphamide, vincristine, and etoposide administered at 10–14-day intervals. This dose-dense approach aims to limit tumor regrowth between cycles while maintaining manageable toxicity [46]. Comparative studies have demonstrated that the COG and COJEC induction strategies yield similar response rates and toxicity profiles, with no significant differences in overall outcomes [47].

During the induction phase, hematopoietic stem cell collection is routinely performed in preparation for subsequent autologous stem cell transplantation (ASCT). In the COG protocols, peripheral blood stem cells are typically harvested after the second chemotherapy cycle, whereas in the rapid COJEC regimen, collection usually occurs following the eighth cycle [48].

Surgical resection of the primary tumor is generally scheduled after four to six cycles of induction chemotherapy, once maximal tumor shrinkage has been achieved. Delaying surgery until this point increases the likelihood of complete or near-complete resection while minimizing operative morbidity. Indeed, the introduction of induction chemotherapy has significantly improved resectability rates, with complete resection rates increasing from approximately 27% to nearly 45% in high-risk patients [49]. Nevertheless, surgical risks remain substantial, particularly in patients with extensive disease or vascular involvement, underscoring the importance of careful multidisciplinary planning.

#### 6.2.2. Consolidation Phase

The consolidation phase follows induction therapy and is designed to eradicate minimal residual disease, thereby reducing the risk of relapse. This phase typically consists of high-dose chemotherapy followed by ASCT, often combined with locoregional radiotherapy. Multiple clinical trials have demonstrated that this approach significantly improves event-free survival compared with chemotherapy alone or no further treatment after induction [50].

High-dose chemotherapy regimens used during consolidation vary by geographic region. In North America, the most commonly employed conditioning regimen consists of carboplatin, etoposide, and melphalan (CEM). In contrast, European protocols more frequently utilize the combination of busulfan and melphalan (Bu/Mel), which has shown comparable or, in some studies, superior efficacy with an acceptable toxicity profile [51]. The choice of conditioning regimen is influenced by institutional preference, prior treatment exposure, and patient-specific factors.

Following hematopoietic recovery after ASCT, radiotherapy is administered to the primary tumor site and, when indicated, to residual metastatic lesions. The standard radiation dose is approximately 21 Gy, delivered in fractionated schedules to minimize toxicity. Advances in radiotherapy techniques, including the use of proton beam therapy, have allowed for improved dose conformality and reduced exposure of surrounding healthy tissues, thereby lowering the risk of long-term treatment-related morbidity without compromising local tumor control [43].

#### 6.2.3. Maintenance Phase

The maintenance phase is designed to eradicate minimal residual disease that persists following induction and consolidation therapy and represents a critical step in reducing the risk of relapse in high-risk neuroblastoma. Over time, several post-consolidation strategies have been developed to improve long-term disease control and event-free survival (EFS).

One of the most significant advances in this phase has been the introduction of differentiation therapy with isotretinoin (13-cis-retinoic acid). This agent promotes terminal differentiation and inhibits the proliferation of residual neuroblastoma cells. Randomized clinical trials have demonstrated that isotretinoin significantly improves EFS in patients who achieve remission after intensive multimodal therapy, establishing it as a cornerstone of maintenance treatment [52].

Building upon this foundation, immunotherapy has emerged as a transformative approach in the management of high-risk neuroblastoma. Monoclonal antibodies targeting the disialoganglioside GD2—an antigen highly expressed on neuroblastoma cells—have demonstrated substantial clinical benefit. Early trials incorporating the chimeric anti-GD2 antibody dinutuximab (ch14.18) in combination with isotretinoin showed significantly improved survival outcomes compared with isotretinoin alone [53].

Subsequent randomized phase III studies further enhanced these results by combining anti-GD2 immunotherapy with immune-stimulatory cytokines, such as granulocyte–macrophage colony-stimulating factor (GM-CSF) and interleukin-2 (IL-2). This multimodal immunotherapeutic approach led to superior event-free survival and overall survival compared with standard therapy, establishing anti-GD2-based immunotherapy as a standard component of maintenance treatment for high-risk neuroblastoma [54].

Despite the demonstrated survival benefit of anti-GD2 monoclonal antibodies, their use is associated with a distinct toxicity profile that requires meticulous supportive care and multidisciplinary management. The most frequent and clinically significant adverse effect is neuropathic pain, resulting from GD2 expression on peripheral nerve fibers [53]. This pain can be severe, particularly during initial infusions, and often necessitates premedication and continuous analgesic strategies, including opioid infusions, gabapentinoids, ketamine, and non-opioid adjuncts. Additional toxicities include capillary leak syndrome, hypotension, fever, hypersensitivity reactions, and gastrointestinal symptoms, which may require aggressive fluid management, vasopressor support, and temporary treatment interruption [53]. Autonomic dysfunction, electrolyte disturbances, and ocular complications such as mydriasis or blurred vision have also been reported and warrant close monitoring [53]. The successful delivery of anti-GD2 immunotherapy, therefore, depends not only on oncologic expertise but also on comprehensive supportive care protocols involving pain specialists, intensive care support when necessary, and experienced nursing teams. Standardized toxicity management algorithms and anticipatory symptom control are essential to ensure treatment adherence, minimize morbidity, and maintain quality of life during maintenance therapy.

Collectively, these advances have transformed the maintenance phase into a critical therapeutic window, shifting the focus from cytotoxic therapy toward immune-mediated disease control and significantly improving long-term outcomes for patients with high-risk neuroblastoma.

### 6.3. Long-Term Toxicities and Survivorship Considerations

The high intensity of multimodal therapy required for intermediate- and high-risk neuroblastoma—including multi-agent chemotherapy, radiotherapy, high-dose chemotherapy with ASCT, and immunotherapy—has led to a growing population of long-term survivors who face a substantial burden of late effects. Recognition and proactive management of these therapy-related toxicities are essential components of comprehensive survivorship care models.

Among the most prevalent long-term complications is ototoxicity, primarily associated with platinum-based chemotherapy (cisplatin and carboplatin). Sensorineural hearing loss occurs in a significant proportion of survivors and may be progressive, bilateral, and irreversible, with profound consequences for language development, academic achievement, and social integration, particularly in younger children. Early audiologic surveillance and timely intervention with hearing support services are therefore critical.

Endocrinopathies represent another major category of late effects, arising from a combination of craniospinal or abdominal radiotherapy, alkylating agents, and total-body exposure during myeloablative conditioning. Survivors are at increased risk for growth hormone deficiency, hypothyroidism, gonadal dysfunction, delayed or precocious puberty, and impaired fertility. These complications may emerge years after treatment completion, underscoring the need for long-term endocrine follow-up extending into adulthood.

Neuroblastoma survivors are also vulnerable to secondary malignancies, particularly therapy-related myelodysplastic syndrome and acute myeloid leukemia following exposure to topoisomerase II inhibitors and alkylating agents, as well as solid tumors arising within irradiated fields. Although relatively uncommon, these secondary cancers carry significant morbidity and mortality and necessitate lifelong vigilance.

Additional late effects include cardiotoxicity related to anthracycline exposure, renal dysfunction, pulmonary impairment, musculoskeletal abnormalities, and persistent neurocognitive deficits. Importantly, the cumulative impact of these physical sequelae often intersects with psychological distress, educational challenges, and reduced quality of life.

Taken together, these risks highlight the imperative for structured, risk-adapted survivorship care models that incorporate multidisciplinary follow-up, early screening for late effects, psychosocial support, and transition planning from pediatric to adult care services. Such models are essential not only to mitigate long-term morbidity but also to optimize functional outcomes and quality of life for survivors of childhood neuroblastoma.

However, beyond the description of persistent neurological sequelae, the potential for functional recovery in neuroblastoma survivors should be considered within the broader framework of brain plasticity. Contemporary neuroscience research has demonstrated that neural recovery after injury is not solely dependent on restitution of damaged networks but also on dynamic hemispheric interactions and the recruitment of compensatory pathways [55]. Functional reorganization may involve the redistribution of cognitive and motor functions across homologous regions, the engagement of alternative neural circuits, and experience-dependent synaptic remodeling. Evidence from pediatric and adult neuroimaging studies indicates that such compensatory mechanisms can support partial or substantial recovery even after early-life neural insults, particularly when rehabilitation occurs during sensitive developmental windows characterized by heightened plasticity [55]. Situating neurological outcomes in this context provides an important physiological rationale for early, sustained, and individualized rehabilitation strategies within survivorship care. Integrating insights from studies on neural compensation and interhemispheric rebalancing further underscores the capacity for adaptive brain reorganization and strengthens the argument for multidisciplinary follow-up models that actively support functional recovery rather than viewing neurological deficits as static end points.

## 7. Future Treatment Options

In recent years, outcomes for patients with high-risk neuroblastoma have improved significantly, largely due to the incorporation of immunotherapy into standard maintenance regimens. These advances have shifted the therapeutic landscape and highlighted the importance of prolonged post-consolidation treatment in achieving durable disease control.

Current research efforts are increasingly focused on extending and optimizing maintenance strategies for patients in first remission, with the goal of further improving long-term survival. Several ongoing clinical trials are evaluating the integration of novel agents into frontline therapy, including the use of ^131I-metaiodobenzylguanidine (MIBG) in patients with MIBG-avid disease during induction or consolidation phases [56].

Parallel to these therapeutic advances, major progress in genomic and molecular profiling has deepened our understanding of the biological heterogeneity of neuroblastoma. High-throughput sequencing approaches, including whole-exome, whole-genome, and transcriptome sequencing, have identified recurrent genetic alterations involving genes such as *ALK*, *PTPN11*, *ATRX*, *MYCN*, and *NRAS*, many of which represent potential therapeutic targets [57,58]. This growing molecular insight has fueled the development of precision medicine strategies aimed at tailoring treatment to the individual tumor’s genetic profile.

Approximately 10% of sporadic and familial high-risk neuroblastomas harbor activating mutations in the *ALK* gene. Targeted inhibition of *ALK* has therefore emerged as a promising therapeutic strategy. Clinical trials evaluating *ALK* inhibitors, such as crizotinib, in patients with *ALK*-mutated neuroblastoma have demonstrated encouraging activity, supporting the integration of molecularly targeted therapies into treatment paradigms for selected patient subsets [59].

Despite the therapeutic promise of molecularly targeted approaches, both *ALK*-directed therapies and *MYCN*-driven strategies are limited by the emergence of treatment resistance, underscoring the need for continued therapeutic refinement. In *ALK*-mutated neuroblastoma, resistance to first-generation *ALK* inhibitors such as crizotinib has been associated with secondary kinase-domain mutations that alter drug binding, as well as *ALK* gene amplification and activation of bypass signaling pathways involving MAPK and *PI3K/AKT* cascades. These adaptive mechanisms can emerge under selective therapeutic pressure and contribute to disease relapse. Such findings provide a strong biological rationale for the development and clinical testing of next-generation *ALK* inhibitors with greater potency and activity against resistance-conferring mutations, as well as for combination strategies that simultaneously target downstream signaling pathways. Similarly, *MYCN*-amplified tumors exhibit intrinsic and acquired resistance mediated by transcriptional plasticity, epigenetic reprogramming, and metabolic adaptation, limiting the efficacy of indirect *MYCN*-targeting approaches. A deeper understanding of these resistance mechanisms is therefore essential to guide rational drug design, optimize combination therapies, and improve the durability of targeted treatments in high-risk neuroblastoma.

Collectively, these advances highlight a paradigm shift toward biologically driven, risk-adapted therapy. Continued integration of molecular diagnostics with novel targeted and immunotherapeutic approaches holds significant promise for improving cure rates while minimizing treatment-related toxicity, ultimately advancing personalized care for children with high-risk neuroblastoma.

Although a growing body of literature has described emerging therapeutic modalities in neuroblastoma—including targeted agents, immunotherapies, cellular therapies, and radiopharmaceuticals—their optimal clinical integration remains an unresolved challenge. Rather than representing competing strategies, these approaches should be viewed as biologically complementary, each addressing distinct aspects of tumor heterogeneity and disease persistence. Targeted therapies, such as *ALK* inhibitors, offer precision against defined oncogenic drivers but are limited by clonal evolution and acquired resistance. Immunotherapeutic strategies, including anti-GD2 monoclonal antibodies and CAR T cells, provide durable immune-mediated control yet face barriers related to antigen heterogeneity, tumor microenvironment–mediated immune suppression, and treatment-related toxicity. Radiopharmaceuticals such as ^131I-MIBG deliver effective cytoreduction in MIBG-avid disease but are constrained by hematologic toxicity and limited applicability in non-avid tumors. An integrative therapeutic paradigm—combining molecular profiling-guided targeted agents with immune-based consolidation and radiopharmaceutical debulking—may therefore be necessary to overcome the limitations of single-modality approaches. Future clinical trials should prioritize rational sequencing, combination strategies, and biomarker-driven patient selection to maximize efficacy while minimizing cumulative toxicity, thereby advancing from descriptive innovation toward truly precision-based care in high-risk and relapsed neuroblastoma.

## 8. Relapsed Neuroblastoma

Relapsed neuroblastoma remains one of the most formidable challenges in pediatric oncology, with historically poor outcomes despite aggressive multimodal therapy. Nevertheless, recent advances in immunotherapy and targeted treatment strategies have begun to improve response rates, particularly in selected patient subgroups.

Adoptive immunotherapy using GD2-targeted CAR T cells has shown encouraging activity, especially in patients with low disease burden. These approaches exploit the high and uniform expression of GD2 on neuroblastoma cells and represent a promising avenue for durable disease control. In parallel, chemo-immunotherapy continues to play a central role in the management of relapsed disease. Combination regimens incorporating conventional cytotoxic agents such as irinotecan and temozolomide together with anti-GD2 monoclonal antibodies—most notably dinutuximab and naxitamab—have demonstrated meaningful response rates and improved disease control in relapsed or refractory settings [60].

Additional therapeutic advances have emerged from targeted approaches. The BEACON-Neuroblastoma trial demonstrated that the addition of the anti–vascular endothelial growth factor (VEGF) monoclonal antibody bevacizumab to chemotherapy significantly improved objective response rates in patients with relapsed disease, supporting the role of angiogenesis inhibition as a complementary strategy in this setting [61].

For patients who respond to re-induction therapy, consolidation remains a critical component of treatment. Targeted radiopharmaceutical therapy using ^131I- MIBG or ^177Lu-DOTATATE has shown efficacy in eradicating residual disease, particularly in MIBG-avid tumors. When feasible, this is followed by high-dose chemotherapy with busulfan and melphalan and subsequent autologous stem cell transplantation, which remains a cornerstone of consolidation therapy in relapsed high-risk neuroblastoma [62].

Together, these evolving multimodal strategies underscore the shift toward personalized, biology-driven approaches aimed at improving outcomes for children with relapsed neuroblastoma while balancing treatment-related toxicity.

## 9. Conclusions

High-risk and relapsed neuroblastoma continue to represent some of the most formidable challenges in pediatric oncology, despite substantial therapeutic advances achieved over recent decades. While multimodal treatment strategies have significantly improved survival, outcomes for these patients remain suboptimal, underscoring the urgent need for continued innovation and refinement of therapeutic approaches.

Large, well-designed multicenter clinical trials are essential to further evaluate the efficacy, safety, and long-term impact of emerging treatment modalities, including immunotherapies, targeted radiopharmaceuticals such as ^131I-MIBG, and advanced cellular therapies such as CAR T cells. At the same time, addressing mechanisms of treatment resistance remains a critical priority, as relapse continues to be the principal cause of mortality in high-risk disease.

Additional challenges include the need to better define and manage the short- and long-term toxicities associated with novel therapies, as well as to ensure equitable access to these often complex and resource-intensive treatments. The financial and logistical burden associated with advanced therapeutics may limit their widespread implementation, particularly in resource-constrained settings.

Advances in genomic and molecular profiling are reshaping the understanding of neuroblastoma biology and offer unprecedented opportunities for precision medicine. The integration of molecularly targeted therapies, biomarker-driven risk stratification, and individualized treatment strategies holds promise for improving survival while minimizing toxicity. Continued translational research and international collaboration will be essential to translate these scientific advances into durable clinical benefit for children affected by this aggressive disease.

This review underscores the evolving complexity of neuroblastoma as a disease that can no longer be approached solely through the lens of short-term survival. By integrating advances in molecular biology, risk stratification, multimodal therapy, and survivorship outcomes, we highlight the need for a more holistic and longitudinal model of care. Persistent challenges—including therapeutic resistance, treatment-related late effects, neurodevelopmental vulnerability, and disparities in access to advanced therapies—remain key barriers to durable cure and optimal quality of life. Addressing these gaps will require coordinated international collaboration, deeper integration of molecular profiling into clinical decision-making, and the development of adaptive treatment and survivorship strategies that evolve alongside the patient. Future progress in neuroblastoma will depend not only on improving cure rates but also on ensuring that survivors transition into adolescence and adulthood with preserved cognitive, physical, and psychosocial health.

## Data Availability

All the available data are included in the manuscript.
